# Biodiversity of extant snails (Gastropoda, Mollusca) in the Pliocene Mountain Spur Natural Reserve (Northern Apennine, Italy)

**DOI:** 10.3897/BDJ.11.e95688

**Published:** 2023-03-21

**Authors:** Federico Plazzi, Guido Pedroni

**Affiliations:** 1 University of Bologna, Department of Biological, Geological and Environmental Sciences, Bologna, Italy University of Bologna, Department of Biological, Geological and Environmental Sciences Bologna Italy; 2 Ente di Gestione per i Parchi e la Biodiversità - Emilia Orientale - Sede Parco Reg.le Corno alle Scale, Bologna, Italy Ente di Gestione per i Parchi e la Biodiversità - Emilia Orientale - Sede Parco Reg.le Corno alle Scale Bologna Italy; 3 World Biodiversity Association, Verona, Italy World Biodiversity Association Verona Italy

**Keywords:** Pliocene Mountain Spur, Tuscan-Emilian Apennine, checklist, molluscan fauna, Gastropoda, Eupulmonata, pulmonate molluscs, subfossils

## Abstract

**Background:**

To date, there is a substantial lack of information about gastropods from the Tuscan-Emilian Apennine in the north of Italy, notwithstanding the availability of detailed and comprehensive literature on this molluscan class. We present a gastropod fauna from the Natural Reserve of the Pliocene Mountain Spur: to our knowledge, this is the first investigation of the extant gastropod fauna in the Tuscan-Emilian Apennine and one of the few in the Apennine's mountain chain as a whole.

**New information:**

We describe a gastropod fauna comprised by 25 species, belonging to 18 genera and 10 families: the general figure which is emerging is an assemblage of European and Mediterranean-European species, with a single Asian contribution. Several shells were collected as embedded in sandy-clayey soils and showed fossilisation traces: therefore, we regard these shells as subfossil samples. Namely, subfossil shells are from the species *Pomatiaselegans*, *Granariafrumentum*, *Retinellaolivetorum*, *Xerolentaobviaobvia*, Oxychiluscf.draparnaudi, *Monachacartusiana* and *Monachacantiana*. The present checklist is the first report in the Tuscan-Emilian Apennine and Emilia-Romagna areas for six taxa: *Morlinaglabraglabra*, *Oxychiliusalliarius*, *Xerosectacespitum*, *Fruticicolafruticum*, *Xerogyraspadae* and *Xerolentaobviaobvia*.

## Introduction

The order Stylommatophora is by far the best-known and largest order of the superorder Eupulmonata (Gastropoda), including approximately 25,000-30,000 terrestrial species in 104 families (Mordan and Wade 2008, Brusca et al. 2016, Doğan et al. 2020, Kosicka et al. 2022). Stylommatophorans are comprised of land snails and slugs. The shell may be completely or partly enveloped by dorsal mantle or may be absent. The crown age of stylommatophorans has been estimated around the Late Cretaceous, about 140 million years ago (Doğan et al. 2020).

Currently, 2,087 species of non-marine gastropods are known to inhabit Europe. Amongst these non-marine gastropod species, as many as 1,834 (87.88%) are eupulmonates, of which only 24 (1.31%) are non-stylommatophorans; in Italy, 345 species belonging to Eupulmonata have been detected (Welter-Schultes 2012). However, it is worth recalling that groups such as Hydrobioidea and related taxa are comprised of very small freshwater snails with really restricted ranges and diffused nomenclatural issues and only expert taxonomists are able to identify such groups that are, therefore, largely underestimated (or absent) in faunal checklists (Welter-Schultes 2012).

**Figure 1.**
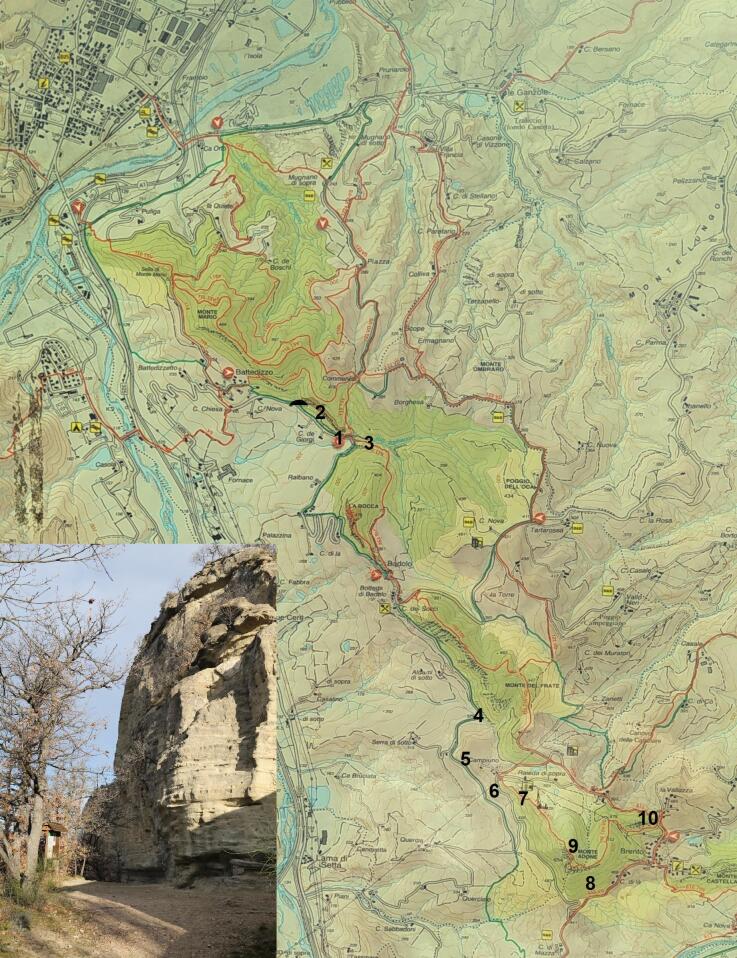
A map of the Pliocene Mountain Spur Area. The Natural Reserve is highlighted in green; sampling localities are shown with numbers 1-10 and refer to those in Table 1 and shown in Fig. 2. Lower left insert shows a typical morphology of the Spur: the sandstone wall of Rocca di Badolo with its large ledge.

The area of the Pliocene Mountain Spur is a system of rocky outcrops of sandstone dating back to the Pliocene, which ranges between 2.58 and 5.33 million years ago (mya). The Pliocene Mountain Spur encompasses the valleys of rivers Setta, Savena, Zena and Idice; it spans over the Tuscan-Emilian Apennines for a length of about 15 km, covering an approximate surface area of 2,628 ha (Fig. 1).

The Natural Reserve of the Pliocene Mountain Spur was created in 2006. It covers a narrower area (around 757 ha), consisting of a small-sized, 8 km-long mountain chain between Sasso Marconi and Brento, from the beginning of the Setta Valley. It is included in the Sites of Community Importance and in the Special Protection Areas (SCI/SPA IT4050012), as well as in the Natura 2000 Network (2628 ha); moreover, it is safeguarded under the Emilia-Romagna Regional Landscape Territorial Plan and the established Faunal Oasis.

The molluscan fauna that we present herein is of relevant interest, since the Northern Apennine has seldom been an object of well-structured malacological studies. Data are lacking for the Natural Reserve presented above; comprehensive publications on molluscan fauna of the Northern Apennines (or of smaller areas within) are limited, the most detailed and in-depth study being a work on the area surrounding Pistoia (Cianfanelli 2009), to which a few other works should be added (Bodon and Cianfanelli 2002, Bodon 2007). Conversely, Northern Apennine areas might turn out to be actually very interesting because of the peculiar morphological and ecological features of the region, including the mountain ridge above the tree line (approximately 2,000 m); lacustrine basins and surrounding habitats; gullies and caverns that may be found in Apennine hills. Indeed, the study of this fauna provided insightful information on gastropod ecology and biogeography.

## Materials and methods

The present work contributes to the current knowledge of extant gastropod molluscs of the Pliocene Mountain Spur, with special reference to the superorder Eupulmonata. Furthermore, we also report subfossil samples, i.e. molluscan (in the present case) samples that did not complete the long and complicated physical/chemical fossilisation process (see, for instance, Gambetta (1934), Cuscani Politi (1988), Lencioni (2006)).

The faunal list and ecology notes are partly taken from previous work (Kerney and Cameron 1979, Boato et al. 1984, Boato et al. 1987, Cossignani and Cossignani 1995, Welter-Schultes 2012, Cossignani and Cossignani 2020).

**Figure 2.**
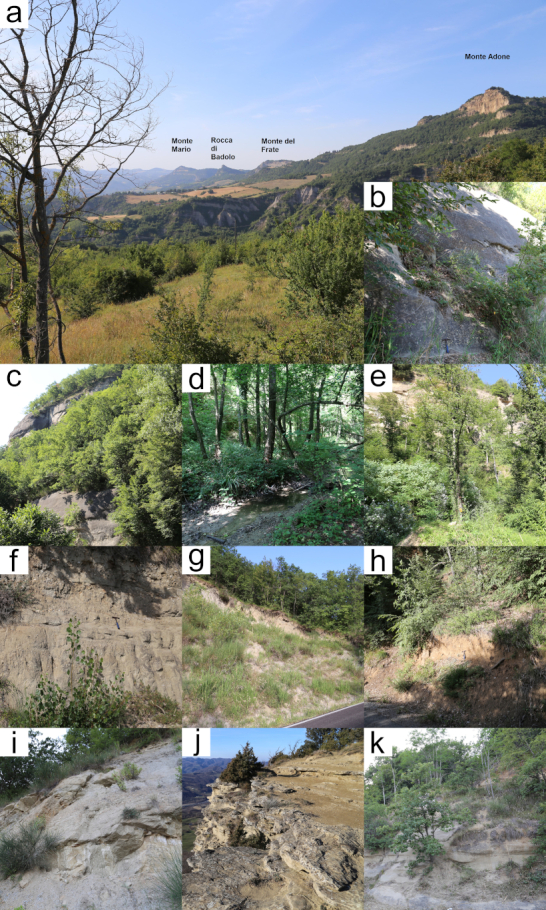
Study area. **a** overview of the Pliocene Mountain Spur; **b** sampling site 1 (Monte Mario); **c** sampling site 2 (slopes of Monte Mario); **d** sampling site 3 (Fosso Raibano); **e** sampling site 4 (base of Monte del Frate); **f** sampling site 5 (road between Monte del Frate and Monte Adone); **g** sampling site 6 (road to Monte Adone, below Campiuno); **h** sampling site 7 (Campiuno); **i** sampling site 8 (Brento); **j** sampling site 9 (top of Monte Adone); **k** sampling site 10 (Brento, towards Pianoro).

Mollusc specimen collection was carried out by exploring several Reserve sites, sampling both from the surface and removing the sandy-clayey sediment derived from erosion, down to a depth of 50-80 cm. Living specimens were collected for all the species of the molluscan fauna presented here; subfossil specimens were also collected for some of them (Table 1). Specimens were collected from different sources: leaf litter, rocky outcrop crevices, lower side of rock fragments, sandy deposit along the paths and at the base of sandstone walls and soil in the proximity of water bodies. Sampling sites are detailed and shown in Table 1 and Fig. 2.

Species determination was carried out on conchological characters, relying on the available malacological literature (e.g., Kerney and Cameron 1979, Cossignani and Cossignani 1995, Cianfanelli 2009, Welter-Schultes 2012, Cossignani and Cossignani 2020).

Shells were deposited and stored in the collection of one of the authors (GP). Each specimen was given a specimen ID; the abbreviation "PMS" stands for "Pliocene Mountain Spur" and each site is followed by the reference number as provided in Table 1.

## Checklists

### Checklist of gastropod molluscs (Littorinimorpha and Eupulmonata) in the Pliocene Mountain Spur Natural Reserve (Northern Apennines, Italy)

#### Gastropoda

Cuvier, 1795

A91B786D-60EF-5272-8D20-5B985AFA7FA8

#### Caenogastropoda

L. R. Cox, 1960

9B2CF0DC-D872-5648-9CC5-E0C416C84B2C

#### Littorinimorpha

Golikov & Starobogatov, 1975

EE39C29D-97DA-5D1B-8C67-29A19AF407B8

#### Littorinoidea

Children, 1834

957FE238-DE45-533A-A311-52D2A07B2734

#### Pomatiidae

Newton, 1891 (1828)

094E0EBE-95D3-5288-BAFC-B0D03EFFA59E

#### Pomatias elegans

(O. F. Müller, 1774)

0B77EDE0-C98B-5D1B-9A6A-EAD5FB57D327

##### Materials

- **Type status:** Other material. **Occurrence:** recordedBy: Pedroni; individualCount: several specimens; occurrenceID: CE507DD9-F489-53F7-9B21-83C963A6A4C9; **Location:** country: Italy; locality: Fosso (Rio) Raibano (3), PMS, Setta Valley; verbatimElevation: 331 m; **Identification:** identifiedBy: Pedroni; **Event:** eventDate: 12.IX.2020
- **Type status:** Other material. **Occurrence:** recordedBy: Pedroni; individualCount: several specimens; occurrenceID: A4C6A5BC-1688-5E46-97DB-C326924CF0C0; **Location:** country: Italy; locality: Monte Mario (1), PMS, Setta Valley; verbatimElevation: 231 m; **Identification:** identifiedBy: Pedroni; **Event:** eventDate: 31.VIII.2020
- **Type status:** Other material. **Occurrence:** recordedBy: Pedroni; individualCount: 3; occurrenceID: EF7C25FA-BA6A-59F7-A04D-EE9C2C9B2701; **Location:** country: Italy; locality: Monte Mario slopes (2), PMS, Setta valley; verbatimElevation: 241 m; **Identification:** identifiedBy: Pedroni; **Event:** eventDate: 17.VIII.2021
- **Type status:** Other material. **Occurrence:** recordedBy: Pedroni; individualCount: several specimens; occurrenceID: 39C40D1D-FCFB-58A7-9E72-B327D7F07050; **Location:** country: Italy; locality: Road to Monte Adone (below Campiuno) (6), PMS, Setta Valley; verbatimElevation: 374 m; **Identification:** identifiedBy: Della Bella & Scarponi; **Event:** eventDate: 30.VIII.2020
- **Type status:** Other material. **Occurrence:** recordedBy: Pedroni; individualCount: 8 + several juveniles; occurrenceID: 8CC927F5-EC56-575F-B9E4-85542DAB8F6C; **Location:** country: Italy; locality: Road to Monte Adone (below Campiuno) (6), PMS, Setta Valley; verbatimElevation: 374 m; **Identification:** identifiedBy: Della Bella & Scarponi; **Event:** eventDate: 28.VIII.2020
- **Type status:** Other material. **Occurrence:** recordedBy: Pedroni; individualCount: 6; occurrenceID: AB47EEA8-8A12-584B-A831-1C7F29575591; **Location:** country: Italy; locality: Near Campiuno, between Monte del Frate and Monte Adone (reddish soil outcrop) (7), PMS, Setta Valley; verbatimElevation: 421 m; **Identification:** identifiedBy: Della Bella & Scarponi; **Event:** eventDate: 30.VIII.2020
- **Type status:** Other material. **Occurrence:** recordedBy: Pedroni; individualCount: 4; occurrenceID: 62D95ECA-5CF8-5EB3-9D6F-810F63F4ED2C; **Location:** country: Italy; locality: Near Campiuno, between Monte del Frate and Monte Adone (7), PMS; verbatimElevation: 421 m; **Identification:** identifiedBy: Pedroni; **Event:** eventDate: 5.IX.2021

##### Notes

Shell thick, ovoidal and grossly conical; spire with 4-5 convex coils; pattern reticulated, ranging from beige to light purple; operculum calcareous or chalky. Some specimens were collected on sandy-clayey slopes that line the road connecting Monte del Frate and Monte Adone, down to a depth of 60 cm. Others were collected at similar depths in the underbrush along a creek (Fosso Raibano); finally, some specimens were collected from the underbrush floor. The species is thermophilic and euryecious; it is widespread and present on calcareous soils, in meadows and underbrush, either uncultivated or cultivated, from hills to plains (Cossignani and Cossignani 1995). Drought periods of several months do not affect survival of this species (Welter-Schultes 2012). Recalling it is a burrowing species, it requires a loose substrate (Kerney and Cameron 1979), where it can dig at least 10 cm, to estivate or hibernate (Welter-Schultes 2012). This species has been described as xerophilic and calcicolous (Boato et al. 1984); moreover, it has been found in the Quaternary dune at Capo Mele (Savona, Italy) as a fossil (Boato et al. 1984).

#### Heterobranchia

Burmeister, 1837

B8335D03-64C8-5342-BBE3-9888C889E127

#### Eupulmonata

Haszprunar & Huber, 1990

6B4BBD4C-2543-5B0B-96B2-2C416042AC9B

#### Stylommatophora

A. Schmidt, 1855

BD2C0271-F45C-5700-83E3-DAB07B0E7C5F

#### Helicina

Rafinesque, 1815

960BA47A-5DB7-5D0B-B920-A6ECEC43A071

#### Chondrinoidea

Steenberg, 1925

3264C19F-D66C-5F08-9B87-823C5D034BB7

#### Chondrinidae

Steenberg, 1925

F4E5141B-6796-5B23-9EA5-FFE0F26CF2AA

#### Granaria frumentum apennina

(Küster, 1847)

BD708271-132C-564A-B5E4-1D720125913D

##### Materials

- **Type status:** Other material. **Occurrence:** recordedBy: Pedroni; individualCount: 1; occurrenceID: EA9A84CB-A72E-558B-9582-89E97979B3A1; **Location:** country: Italy; locality: Road to Monte Adone (below Campiuno) (6), PMS, Setta Valley; verbatimElevation: 374 m; **Identification:** identifiedBy: Pedroni; **Event:** eventDate: 31.XII.2018

##### Notes

Shell cylindrical, short, stubby, brownish; cervical callus thick; grooves thin and regular; normally the aperture shows eight folds and four palatal lamellae, visible from outside (Nardi and Niero 2013). A only single specimen was collected beside the road, at the surface, near Campiuno.

#### Granaria frumentum illyrica

(Rossmässler, 1835)

A93EF1A3-2523-51E1-A882-D484C02DDD0E

##### Materials

- **Type status:** Other material. **Occurrence:** recordedBy: Pedroni; individualCount: 2; occurrenceID: 1E28A32E-33C6-5EC1-AAFA-8E71CC9E57A6; **Location:** country: Italy; locality: Road to Monte Adone (below Campiuno) (6), PMS, Setta Valley; verbatimElevation: 374 m; **Identification:** identifiedBy: Della Bella & Scarponi; **Event:** eventDate: 28.VIII.2020
- **Type status:** Other material. **Occurrence:** recordedBy: Pedroni; individualCount: 1; occurrenceID: 0671FEB1-7A7A-5816-90F4-ECE0142B4252; **Location:** country: Italy; locality: Near Campiuno, between Monte del Frate and Monte Adone (7), PMS, Setta Valley; verbatimElevation: 421 m; **Identification:** identifiedBy: Della Bella & Scarponi; **Event:** eventDate: 31.XII.2020
- **Type status:** Other material. **Occurrence:** recordedBy: Pedroni; individualCount: 1; occurrenceID: 39CBB887-B673-50CB-AFEE-A81DD289725C; **Location:** country: Italy; locality: Near Campiuno, between Monte del Frate and Monte Adone (7), PMS, Setta Valley; verbatimElevation: 421 m; **Identification:** identifiedBy: Pedroni; **Event:** eventDate: 5.IX.2021

##### Notes

Shell cylindrical, short, stubby, brownish. Only one specimen out of four was collected at a depth of about 40 cm, near Campiuno on reddish soil. The subspecies is typically found on calcareous soils in the underbrush litter, as well as at the base of rocks, in sunny calcareous areas, on screes and walls (Cossignani and Cossignani 1995, Kerney and Cameron 1979, Welter-Schultes 2012, Nardi and Niero 2013). The subspecies can be found up to 1,300 m a.s.l. in Switzerland (Welter-Schultes 2012).

#### Granaria variabilis

(Draparnaud, 1801)

5A49E3AB-FE96-56E0-ACA8-A886AFCC58E0

##### Materials

- **Type status:** Other material. **Occurrence:** recordedBy: Pedroni; individualCount: 2; occurrenceID: CBC717D2-4AD1-5585-BF5A-0B5F0FFE9AD8; **Location:** country: Italy; locality: Road to Monte Adone (below Campiuno) (6), PMS, Setta Valley; verbatimElevation: 374 m; **Identification:** identifiedBy: Della Bella & Scarponi; **Event:** eventDate: 31.XII.2018

##### Notes

Shell cylindrical, short, stubby, brownish. Specimens were collected on the sandy underbrush soil, at the surface. The species inhabits environments with calcareous soils, provided that herbaceous or shrubby vegetation is present (Cossignani and Cossignani 1995, Nardi and Niero 2013). *Granariavariabilis* is a xerothermophilic species that dwells also on dry walls, from the sea level to 1,600 m a.s.l. (Kerney and Cameron 1979, Boato et al. 1984, Welter-Schultes 2012).

#### Pupilloidea

W. Turton, 1831

21514B4F-20B6-5250-B8CC-067F68496C8A

#### Enidae

B. B. Woodward, 1903 (1880)

759E112B-FACA-542A-B46A-F248211081A3

#### Jaminia quadridens quadridens

(O. F. Müller, 1774)

6FF7C379-C431-563D-8D4D-01AC2DCDBF98

##### Materials

- **Type status:** Other material. **Occurrence:** recordedBy: Pedroni; individualCount: 3; occurrenceID: BEE2EDF3-FBAE-509A-988E-65D64E33B693; **Location:** country: Italy; locality: Road to Monte Adone (below Campiuno) (6), PMS, Setta Valley; verbatimElevation: 374 m; **Identification:** identifiedBy: Della Bella & Scarponi; **Event:** eventDate: 28.VIII.2020
- **Type status:** Other material. **Occurrence:** recordedBy: Pedroni; individualCount: 2; occurrenceID: A9D5E691-3782-5561-A659-62F62F047987; **Location:** country: Italy; locality: Road between Monte del Frate and Monte Adone (5), PMS, Setta Valley; verbatimElevation: 336 m; **Identification:** identifiedBy: Della Bella & Scarponi; **Event:** eventDate: 31.XII.2018
- **Type status:** Other material. **Occurrence:** recordedBy: Pedroni; individualCount: 1; occurrenceID: DE4469CE-CD68-55C6-AEAD-44C7ACB4B8EB; **Location:** country: Italy; locality: Near Campiuno, between Monte del Frate and Monte Adone (7), PMS, Setta Valley; verbatimElevation: 421 m; **Identification:** identifiedBy: Pedroni; **Event:** eventDate: 5.IX.2021

##### Notes

Shell small, grossly stubby and cylindrical; spire with 7-9 coils; four teeth are visible in the aperture, at different levels of development; peristome white; colour beige. Samples were collected on sandy soils beside the road to Monte Adone, at the surface, as well as in underbrush upstream to the road, near Campiuno. This subspecies inhabits calcareous soils and lives on grass and shrubs in sunny areas, as well as under rocks, in crevices and on screes (Cossignani and Cossignani 1995). Calcareous soils are populated by this subspecies from the plain to the mountain levels, in xeric environments, less commonly on grassy and shrubby vegetation (Kerney and Cameron 1979). It tolerates extensive grazing and can be found in sheep and goat pastures, up to 2,400 m a.s.l. in the Alps (Welter-Schultes 2012). It was also reported as a Pleistocene fossil from Varazze (Savona, Italy; Boato et al. (1984)).

#### Helicoidea

Rafinesque, 1815

A449CD8D-4E46-5071-8ABB-E3A33FCE8C23

#### Hygromiidae

Tryon, 1866

96E99F52-BAC8-5790-B466-ECD868D9A97F

#### Euomphalia strigella strigella

(Draparnaud, 1801)

A90282E3-3A36-56F5-B013-DDA4F95B4548

##### Materials

- **Type status:** Other material. **Occurrence:** recordedBy: Pedroni; individualCount: 1; occurrenceID: C6EBFEE0-B361-5441-B9D3-6CFF0076345B; **Location:** country: Italy; locality: Monte Adone (top) (9), PMS; verbatimElevation: 654 m; **Identification:** identifiedBy: Pedroni; **Event:** eventDate: 13.XII.2021

##### Notes

Shell solid, globular, ranging from brown to reddish-brown; border of the aperture white; the last part of the last coil leans towards the aperture. Adult specimens of this subspecies inhabit moderately open and sunny habitats, avoiding excessive moisture, as is the case for the top of Monte Adone. It is possible, even if uncommon, to find individuals of this subspecies in deciduous woods, either in the leaf litter or on trunks or in hedgerows and scrub (Kerney and Cameron 1979, Cossignani and Cossignani 1995, Girod 2011). It lives in shrubs, between leaves and semi-dry meadows at sunny slopes, up to 1,600 m a.s.l. (Welter-Schultes 2012).

#### Monacha cantiana

(Montagu, 1803)

927C6FA7-DB90-5597-B0E5-4EE9E3C522EC

##### Materials

- **Type status:** Other material. **Occurrence:** recordedBy: Pedroni; individualCount: 2; occurrenceID: 5CEE747A-6604-5E17-A809-4BABAB38D985; **Location:** country: Italy; locality: Road between Monte del Frate and Monte Adone (5), PMS, Setta Valley; verbatimElevation: 336 m; **Identification:** identifiedBy: Della Bella & Scarponi; **Event:** eventDate: 31.XII.2018
- **Type status:** Other material. **Occurrence:** recordedBy: Pedroni; individualCount: 9 + several juveniles; occurrenceID: FE561B99-CB3C-5930-9EC7-638BBA0880AC; **Location:** country: Italy; locality: Road to Monte Adone (below Campiuno) (6), PMS, Setta Valley; verbatimElevation: 374 m; **Identification:** identifiedBy: Della Bella & Scarponi; **Event:** eventDate: 28.VIII.2020

##### Notes

Shell globular and thin, with a maximum of six coils; globally whitish and translucent, but the last coil is reddish around the peristome; umbilicus evident, but not completely open. Some specimens were collected down to a depth of 60 cm, below Campiuno. The species inhabits open environments, typically near water bodies and at lower elevations (Boato et al. 1984, Cossignani and Cossignani 1995, Cossignani and Cossignani 2020). It is not found in woods, rather it lives in roadside verges, hedges (Kerney and Cameron 1979), railways, dunes and well-drained calcareous soils (Welter-Schultes 2012).

#### Monacha cartusiana

(O. F. Müller, 1774)

D180A2CC-F85C-53DF-B76E-C4641E4FCEA0

##### Materials

- **Type status:** Other material. **Occurrence:** recordedBy: Pedroni; individualCount: 2; occurrenceID: 513F70C9-75C0-54D6-9324-53E95FC05D31; **Location:** country: Italy; locality: Monte del Frate (at the base of the mountain in a moist dell) (4), PMS, Setta Valley; verbatimElevation: 335 m; **Identification:** identifiedBy: Della Bella & Scarponi; **Event:** eventDate: 28.VIII.2020

##### Notes

Shell delicate, whitish, sometimes beige; peristome edge shows a thin, brown-reddish band; more flattened than *M.cantiana*. Specimens were collected near to the high sandstone outcrops of Monte del Frate, at a depth of approximately 50 cm. This is a thermophilic species with a wide distribution, inhabiting meadows, cultivated fields, gardens, vineyards, roadsides and ruins (Cossignani and Cossignani 1995, Girod 2011, Welter-Schultes 2012); it also tolerates sheep or cattle grazing in grasslands (Welter-Schultes 2012). It is common to find this species even at hill elevations, albeit it becomes rare above 700 m (Kerney and Cameron 1979, Boato et al. 1984, Welter-Schultes 2012).

#### Monacha martensiana

(Tiberi, 1869)

0082DF0A-AED3-5906-8884-1B160F77B3A6

##### Materials

- **Type status:** Other material. **Occurrence:** recordedBy: Pedroni; individualCount: 1 + 1 juvenile; occurrenceID: E714C6A5-E7D5-5D89-B9C8-65CC8D744B16; **Location:** country: Italy; locality: Near Campiuno, between Monte del Frate and Monte Adone (7), PMS, Setta Valley; verbatimElevation: 421 m; **Identification:** identifiedBy: Pedroni; **Event:** eventDate: 5.IX.2021

##### Notes

Shell ranging from whitish to cream, translucent, similar to other species of the genus *Monacha*, but the shell is more flattened. The species dwells in habitats with grassy vegetation, such as meadows, on calcareous substrates (Cossignani and Cossignani 1995, Welter-Schultes 2012).

#### Monachoides incarnatus

(O. F. Müller, 1774)

F11CEBE3-B09B-5811-B645-2AB2E75E0AB6

##### Materials

- **Type status:** Other material. **Occurrence:** recordedBy: Pedroni; individualCount: 4; occurrenceID: 7E444BCE-95AC-5687-BA9D-33DF51218C30; **Location:** country: Italy; locality: Near Campiuno, between Monte del Frate and Monte Adone (7), PMS; verbatimElevation: 421 m; **Identification:** identifiedBy: Pedroni; **Event:** eventDate: 5.IX.2021

##### Notes

Shell ranging from yellowish to brown-reddish, translucent; microsculpture reticulated, regular, very fine; aperture edge is normally reddish, with a whitish band inside; umbilicus very narrow, but open. This thermophilic subspecies (Girod 2011) can be found in moist environments, woods and hedges, in sunny areas, up to an elevation of 1,500 m a.s.l., especially when shrubby vegetation is present (Kerney and Cameron 1979, Cossignani and Cossignani 1995, Girod 2011). Juveniles climb up plants and feed on rotting vegetation, while adults feed on dead leaves of plants near water bodies (Welter-Schultes 2012).

#### Helicidae

Rafinesque, 1815

A7EA4DDB-0F40-5040-A98E-086A06417335

#### Cornu aspersum

(O. F. Müller, 1774)

54546044-505A-5F36-9B1D-802FA38810DF

##### Materials

- **Type status:** Other material. **Occurrence:** recordedBy: Pedroni; individualCount: 1; occurrenceID: 387D7323-CCBB-5FC8-8E7F-96F767117F06; **Location:** country: Italy; locality: Slopes of Monte Mario (2), PMS, Setta Valley; verbatimElevation: 231 m; **Identification:** identifiedBy: Torchi & Pedroni; **Event:** eventDate: 31.VIII.2020

##### Notes

Background colour from yellowish to beige to greenish; normally 1-5 spiral brown bands are present, well evident, with yellow or white grooves; peristome white, the edge being more or less folded outside; umbilicus closed in adults. The single specimen was collected in the underbrush, at the surface, below the walls of Monte Mario. This species inhabits natural meadows, shrubs, dunes, cultivated fields, as well as gardens (Cossignani and Cossignani 1995, Welter-Schultes 2012). It is often a garden pest and has been artificially dispersed across Europe (Kerney and Cameron 1979, Welter-Schultes 2012).

#### Helix cincta

O. F. Müller, 1774

7DAB092E-FD39-5935-8DF4-74A1B45B8AC8

##### Materials

- **Type status:** Other material. **Occurrence:** recordedBy: Pedroni; individualCount: several specimens; occurrenceID: 2EF8E4CE-FFA0-5A46-9895-A5FFF4248C7F; **Location:** country: Italy; locality: Monte Mario (1), PMS, Setta Valley; verbatimElevation: 231 m; **Identification:** identifiedBy: Pedroni; **Event:** eventDate: 31.VIII.2020
- **Type status:** Other material. **Occurrence:** recordedBy: Pedroni; individualCount: 3; occurrenceID: 8F341085-DAEA-5669-9C49-C56931C66206; **Location:** country: Italy; locality: Near Brento (8), PMS, Setta Valley; verbatimElevation: 436 m; **Identification:** identifiedBy: Della Bella & Scarponi; **Event:** eventDate: 28.VIII.2020

##### Notes

Colour globally brown; shape globular; suture shallow; whorls thick and rounded; umbilicus open. Specimens were collected beside the road to Monte Adone-Brento, at the surface. This species inhabits grassy habitats, rocky areas and woodlands (Cossignani and Cossignani 1995). It can burrow in the soil and remain burrowed for long periods: in Crete, it appears for only a few days in a year at the first winter rainfall (Welter-Schultes 2012).

#### Helix ligata

O. F. Müller, 1774

72EE6D9B-1B55-5300-8423-76AA24DA26B6

##### Materials

- **Type status:** Other material. **Occurrence:** recordedBy: Pedroni; individualCount: 2; occurrenceID: 119F39EC-11D6-54FC-AB64-D7C1D7F458D6; **Location:** country: Italy; locality: Slopes of Monte Mario (2), PMS, Setta Valley; verbatimElevation: 241 m; **Identification:** identifiedBy: Pedroni; **Event:** eventDate: 31.VIII.2020

##### Notes

The shell is highly variable; typically, it is large, ranging from beige to brown in colour. Specimens were caught in a sandy underbrush, at the surface. This species is common in dense vegetation along water bodies, as well as in the underbrush, in mountain areas from Northern Apennine to Calabria, sometimes over the timberline (Cossignani and Cossignani 1995, Welter-Schultes 2012).

#### Geomitridae

C. R. Boettger, 1909

27FEA086-4F62-5679-8097-424F9E30FA87

#### Candidula unifasciata unifasciata

(Poiret, 1801)

56525CDA-4DEA-5F7B-97A7-EC8543B5F12C

##### Materials

- **Type status:** Other material. **Occurrence:** recordedBy: Pedroni; individualCount: 2; occurrenceID: DA8D1352-57EA-54A3-BABE-446817C4E7C5; **Location:** country: Italy; locality: Slopes of Monte Mario (2), PMS, Setta Valley; verbatimElevation: 241 m; **Identification:** identifiedBy: Pedroni; **Event:** eventDate: 17.VIII.2021

##### Notes

Shell medium-sized to small; ranging from white to grey, with a dark brown band and other patterns (bands or spots) on the lower region; 5-6 coils are present; peristome thick, sometimes with teeth; umbilicus narrow. Specimens were collected in the underbrush, close to the sandstone walls of Monte Mario, at the surface. Individuals of this subspecies perfer dry environments between rocks and rocky outcrops, dry meadows and walls (Cossignani and Cossignani 1995, Welter-Schultes 2012, Cossignani and Cossignani 2020) or, more generically, open habitats and areas where grasses were somewhat scraped, from the mountain plain to the vegetation limit in high altitude plain, up to 2,400 m (Kerney and Cameron 1979, Welter-Schultes 2012). This subspecies is also known as a Pleistocene fossil in the Verezzi area (Savona, Italy; Boato et al. (1984)).

#### Cernuella neglecta

(Draparnaud, 1805)

4B9B8F1A-D898-5608-9D00-4493F170106B

##### Materials

- **Type status:** Other material. **Occurrence:** recordedBy: Pedroni; individualCount: 2; occurrenceID: 2A416867-0A6A-5CA9-ABE8-00E2D7658DC5; **Location:** country: Italy; locality: Road to Monte Adone (near Campiuno) (6), PMS, Setta Valley; verbatimElevation: 374 m; **Identification:** identifiedBy: Della Bella & Scarponi; **Event:** eventDate: 28.VIII.2020
- **Type status:** Other material. **Occurrence:** recordedBy: Pedroni; individualCount: 4; occurrenceID: 008BAE09-14E1-5152-9398-3455C6FCADE9; **Location:** country: Italy; locality: Near Brento, (10), PMS, Savena Valley; verbatimElevation: 428 m; **Identification:** identifiedBy: Pedroni; **Event:** eventDate: 5.IX.2021

##### Notes

Shell small, with white and brown stripes; shape somewhat flattened; umbilicus well open. Specimens were collected on sandy-clayey slopes in front of the road between Monte del Frate and Monte Adone, at the surface. Normally, this species lives in roadsides and screes, on coastal dunes, as well as in arid grasslands (Kerney and Cameron 1979, Cossignani and Cossignani 1995, Cossignani and Cossignani 2020). *Cernuellaneglecta* climbs herbal plants and trunks to estivate (Welter-Schultes 2012). The range characterisation of this species is hampered by misidentification in literature (Welter-Schultes 2012).

#### Cernuella virgata

(Da Costa, 1778)

C340585B-4623-5E9C-A0C4-41ABB737B6CF

##### Materials

- **Type status:** Other material. **Occurrence:** recordedBy: Pedroni; individualCount: 1; occurrenceID: 3003A40D-0E5C-50E9-87AE-6EEB0E3252FF; **Location:** country: Italy; locality: Fosso Raibano, Raibano Valley (3), PMS, Setta Valley; verbatimElevation: 331 m; **Identification:** identifiedBy: Pedroni; identificationQualifier: cf. virgata; **Event:** eventDate: 12.IX.2020

##### Notes

Shell with 5-7 coils, variable in colour and shape, with or without bands; umbilicus open; peristome brown in adults. The specimen was collected along the path in the underbrush through the small Raibano Valley, at the surface. The species inhabits dry, open places: arid meadows, ruderal habitats, hedgerows and grassland (Kerney and Cameron 1979). *Cernuellavirgata* also lives on dunes (Kerney and Cameron 1979): in Romagna, specimens were collected on sandy dunes in the Rimini (Italy) area (Cossignani and Cossignani 1995). This species can live at roadsides, in harvested fields and railways; it ascends vertical walls and attaches to tall plants to escape bottom heat (Welter-Schultes 2012). In Italy, this taxon is highly polymorphic, both in terms of shell structure and genital morphology, so that doubt was cast whether it is a single species or a species complex (Boato et al. 1984).

#### Xerogyra spadae

(Calcara, 1845)

1FFFA285-6759-5CA4-998E-779D07B57CA0

##### Materials

- **Type status:** Other material. **Occurrence:** recordedBy: Pedroni; individualCount: 1; occurrenceID: 05D69269-1C3E-5C29-96A6-5182F7611BF9; **Location:** country: Italy; locality: Slopes of Monte Mario (2), PMS, Setta Valley; verbatimElevation: 241 m; **Identification:** identifiedBy: Pedroni; **Event:** eventDate: 17.VIII.2021

##### Notes

The specimen was collected in the underbrush below the walls of Monte Mario, at the surface. This species typically inhabits cool, moist underbrushes, in rocky grasslands, even at high elevation on the Apennine Mountains (Cossignani and Cossignani 1995, Welter-Schultes 2012). This is the first report for the Northern Apennine and Emilia-Romagna areas.

#### Xerolenta obvia obvia

(Menke, 1828)

FFB7EFBD-B58C-5247-8D41-DEA1703A324E

##### Materials

- **Type status:** Other material. **Occurrence:** recordedBy: Pedroni; individualCount: 5; occurrenceID: FDDD1A48-4DA9-57AB-8219-DE4B3F193DDD; **Location:** country: Italy; locality: Monte Mario (1), PMS, Setta Valley; verbatimElevation: 231 m; **Identification:** identifiedBy: Pedroni; **Event:** eventDate: 31.VIII.2020
- **Type status:** Other material. **Occurrence:** recordedBy: Pedroni; individualCount: 2; occurrenceID: C232715D-7DF3-5EAB-A4B1-29665DE088EF; **Location:** country: Italy; locality: Near Brento, (10), PMS, Savena Valley; verbatimElevation: 428 m; **Identification:** identifiedBy: Pedroni; **Event:** eventDate: 5.IX.2021

##### Notes

Shell thick, smooth, medium-sized and flattened; 5-6 coils in adults; background colour white or white-yellowish, with variable bands, ranging from dark brown to black. Some specimens were collected below the walls of Monte Mario at a depth of about 40 cm. The subspecies is typical of xerothermophilic habitats, such as arid meadows (Cossignani and Cossignani 1995), but also dunes, vineyards, ruins, roadsides and railway dams (Welter-Schultes 2012). It is more active in winter than in summer, when it is active only at rainfalls (Welter-Schultes 2012). It attains up to 2,000 m in the Alps (Kerney and Cameron 1979); this is the first report for the Northern Apennine and Emilia-Romagna areas.

#### Xerosecta cespitum

(Draparnaud, 1801)

1835C582-A869-5ACD-800B-3BC3E29FAD9E

##### Materials

- **Type status:** Other material. **Occurrence:** recordedBy: Pedroni; individualCount: 2; occurrenceID: A584CEB2-3F7E-585C-AA1D-449296410568; **Location:** country: Italy; locality: Road between Monte del Frate and Monte Adone (5), PMS, Setta Valley; verbatimElevation: 336 m; **Identification:** identifiedBy: Pedroni; **Event:** eventDate: 12.VI.2022

##### Notes

Shell medium-sized, very variable, ranging from grey to yellow, with variable bands and spots; 5-6 flattened coils are present; peristome whitish or reddish; umbilicus large. The species inhabits meadows, ruderal habitats, roadsides and margins of arable fields (Cossignani and Cossignani 1995, Welter-Schultes 2012, Cossignani and Cossignani 2020). It is common near coasts and in valleys, but it can inhabit sunny mountain slopes in rocky areas in Italy (Welter-Schultes 2012). This is the first report for the Tuscan-Emilian Apennine and Emilia-Romagna areas.

#### Helicodontidae

Kobelt, 1904

D6755BCC-0FB8-5DF5-9247-99358C5B9889

#### Helicodonta obvoluta obvoluta

(O. F. Müller, 1774)

6921004C-CD4B-577F-BDEE-F975068329D5

##### Materials

- **Type status:** Other material. **Occurrence:** recordedBy: Pedroni; individualCount: 1; occurrenceID: E428237B-B8A9-5F14-ACAE-0B5723E39376; **Location:** country: Italy; locality: Monte del Frate (4), PMS, Setta Valley; verbatimElevation: 335 m; **Identification:** identifiedBy: Pedroni; **Event:** eventDate: 5.IX.2021

##### Notes

Shell medium-sized to small, hairy; umbilicus open; ranging from dark brown to brown; flattened above; peristome flattened; lips somewhat thick, white, with a callus in the aperture with three lobes. This subspecies is found in forests under fallen branches, leaf litter and between stones, usually on calcareous substrate; in the Alps, it may reach 1,900 m, but is rarely found above 1,500 m (Kerney and Cameron 1979, Giusti et al. 1985, Welter-Schultes 2012). It may weakly climb up tree trunks and hibernates in rotting wood logs (Welter-Schultes 2012). Hairs on the shell can reach 1 mm in length and are usually permanent (Welter-Schultes 2012).

The collection site of the present specimen is of particular interest, at the base of sandstone outcrops in a small, moist dell with abundant vegetation - a site which may represent a refugial micro-environment with respect to ecological features of this subspecies.

#### Camaenidae

Pilsbry, 1895

E9DEFC32-7315-5B47-8B6E-496E2BB55EA1

#### Fruticicola fruticum

(O. F. Müller, 1774)

D8990D0E-A341-5F51-A166-79C9B1D8FA03

##### Materials

- **Type status:** Other material. **Occurrence:** recordedBy: Pedroni; individualCount: 1; occurrenceID: 95568C74-02E6-5D98-9204-64145558F19D; **Location:** country: Italy; locality: Fosso Raibano, Raibano Valley (3), PMS, Setta Valley; verbatimElevation: 231 m; **Identification:** identifiedBy: Pedroni; **Event:** eventDate: 12.IX.2020
- **Type status:** Other material. **Occurrence:** recordedBy: Pedroni; individualCount: 3; occurrenceID: 0CFDFB44-BDD2-5981-923C-7EDA1DF0FF9F; **Location:** country: Italy; locality: Near Brento (10), PMS, Savena Valley; verbatimElevation: 428 m; **Identification:** identifiedBy: Pedroni; **Event:** eventDate: 5.IX.2021

##### Notes

Shell medium-sized, globular; umbilicus deep; ranging from white to brown in colour. The specimen was collected in the underbrush, at the surface. The species inhabits open meadows, as well as wood leaf litter, edges of woods and bushes in moist habitats (Cossignani and Cossignani 1995), while it is normally absent from open, sunny sites, since it is sensitive to drought (Kerney and Cameron 1979, Welter-Schultes 2012). This species was also found in the Padan Plain (Boato et al. 1984). It feeds on dead leaves, herbs and mushrooms (Welter-Schultes 2012). This is the first report for the Tuscan-Emilian Apennine and Emilia-Romagna areas.

#### Gastrodontoidea

Tryon, 1866

B790D173-1ABD-50E1-9C8F-F8F904BF0C9A

#### Oxychilidae

Hesse, 1927 (1879)

6B527681-06BA-5029-903F-B1B2873C9F2A

#### Morlina glabra glabra

(Rossmässler, 1835)

95EDE7E5-AE49-56A7-A726-67389C79BD52

##### Materials

- **Type status:** Other material. **Occurrence:** recordedBy: Pedroni; individualCount: 1; occurrenceID: 129A9E1F-8AB6-540C-AAFD-C30C48F3D477; **Location:** country: Italy; locality: Slopes of Monte Mario (2) PMS, Setta Valley; verbatimElevation: 241 m; **Identification:** identifiedBy: Pedroni; **Event:** eventDate: 31.VIII.2020

##### Notes

Shell with five coils; diameter of about 14 mm; deep sutures. The single specimen of *M.g.glabra* was collected in a sandy underbrush below the walls of Monte Mario. Normally, this subspecies is found in underbrush, rocks and screes on open hillsides, as well as in biotopes in front of caves and in open, moist environments, but also leaf litter of deciduous and coniferous forests (Kerney and Cameron 1979, Cossignani and Cossignani 1995, Welter-Schultes 2012). It is mailny montane and can reach up to 1,850 m a.s.l. in Switzerland (Kerney and Cameron 1979). It feeds on plants and juvenile snails (Welter-Schultes 2012). This is the first report for the Northern Apennine and Emilia-Romagna areas.

#### Oxychilus alliarius

(J. S. Miller, 1822)

D10CB53D-C6AA-5D75-B5F7-AF7C52157D19

##### Materials

- **Type status:** Other material. **Occurrence:** recordedBy: Pedroni; individualCount: 1; occurrenceID: CE90D943-667C-5BB7-872B-6E30967CEECC; **Location:** country: 241 m; locality: Slopes of Monte Mario (2), PMS, Setta Valley; verbatimElevation: 241 m; **Identification:** identifiedBy: Pedroni; **Event:** eventDate: 31.VIII.2020

##### Notes

Shell small, yellow-brown, lucent, flattened, with very few, packed coils. The specimen was collected in the underbrush. This species inhabits the leaf litter in broad-leaved, deciduous forests, as well as woods, fields, rocks and gardens; rarely it also inhabits acidic places, such as conifer plantations (Kerney and Cameron 1979, Cossignani and Cossignani 1995). It can also be found at water margins and in cultivated areas with moist meadows (Welter-Schultes 2012). This is the first report for the Tuscan-Emilian Apennine and Emilia-Romagna areas.

#### Oxychilus draparnaudi

(H. Beck, 1837)

1209CED2-D71F-5DC2-8353-42DBBE29FA99

##### Materials

- **Type status:** Other material. **Occurrence:** recordedBy: Pedroni; individualCount: 2; occurrenceID: E94F290E-6777-5EBE-A1C9-EDA1433C6C8F; **Location:** country: Italy; locality: Near Brento (8), PMS, Setta Valley; verbatimElevation: 436 m; **Identification:** identifiedBy: Della Bella & Scarponi; identificationQualifier: cf. draparnaudi; **Event:** eventDate: 28.VIII.2020

##### Notes

The shell is similar to *O.alliarius*, but the colour is darker. Both specimens were collected in the wood upstream to the road to Monte Adone-Brento, at a depth of 40-50 cm. This species inhabits the leaf litter in broad-leaved, deciduous forests; caves, even in depth; vegetation and rocky outcrops with a suitable level of moisture and sheltering (Boato et al. 1984, Cossignani and Cossignani 1995). It is also common in gardens, urban waste ground, roadside rubbish, compost heaps and greenhouses (Kerney and Cameron 1979, Welter-Schultes 2012). *O.draparnaudi* feeds on earthworms, juvenile slugs, juvenile snails, even cat and dog food (Welter-Schultes 2012). Fossils from genus *Oxychilus* were found in Quaternary sites near to Pietra Ligure and Capo Mele (Savona, Italy; Boato et al. 1984).

#### Gastrodontidae

Tryon, 1866

72E45F32-A5A9-5E67-9473-93366F59B368

#### Retinella olivetorum olivetorum

(Gmelin, 1791)

41E35803-0713-5FE9-B3D7-9EF33BC6581A

##### Materials

- **Type status:** Other material. **Occurrence:** recordedBy: Pedroni; individualCount: 4; occurrenceID: B1B7282D-479F-55B9-A4EA-83D9614CDEAA; **Location:** country: Italy; locality: Fosso Raibano, Raibano Valley (3), PMS, Setta Valley; verbatimElevation: 231 m; **Identification:** identifiedBy: Pedroni; **Event:** eventDate: 12.IX.2020
- **Type status:** Other material. **Occurrence:** recordedBy: Pedroni; individualCount: 1; occurrenceID: 690FA42A-CD0B-55E2-8DD8-28760ED2569E; **Location:** country: Italy; locality: Road to Monte Adone (below Campiuno) (6), PMS, Setta Valley; verbatimElevation: 374 m; **Identification:** identifiedBy: Della Bella & Scarponi; **Event:** eventDate: 28.VIII.2020

##### Notes

Shell yellowish-brown, dextral, medium-sized, thin, almost transparent, globular, with 5-6 coils; sutures well evident; umbilicus large, deep, funnel-shaped; peristome unfolded and thin. Some specimens were collected at a depth of up to 50 cm along the Raibano Valley path. Normally, *R.o.olivetorum* inhabits shady hill and mountain meadows in chestnut and olive woods (Cossignani and Cossignani 1995, Welter-Schultes 2012, Cossignani and Cossignani 2020). It can survive drought inside the soil and appears only during and after longer rainfalls (Welter-Schultes 2012).

## Discussion

**Figure 3.**
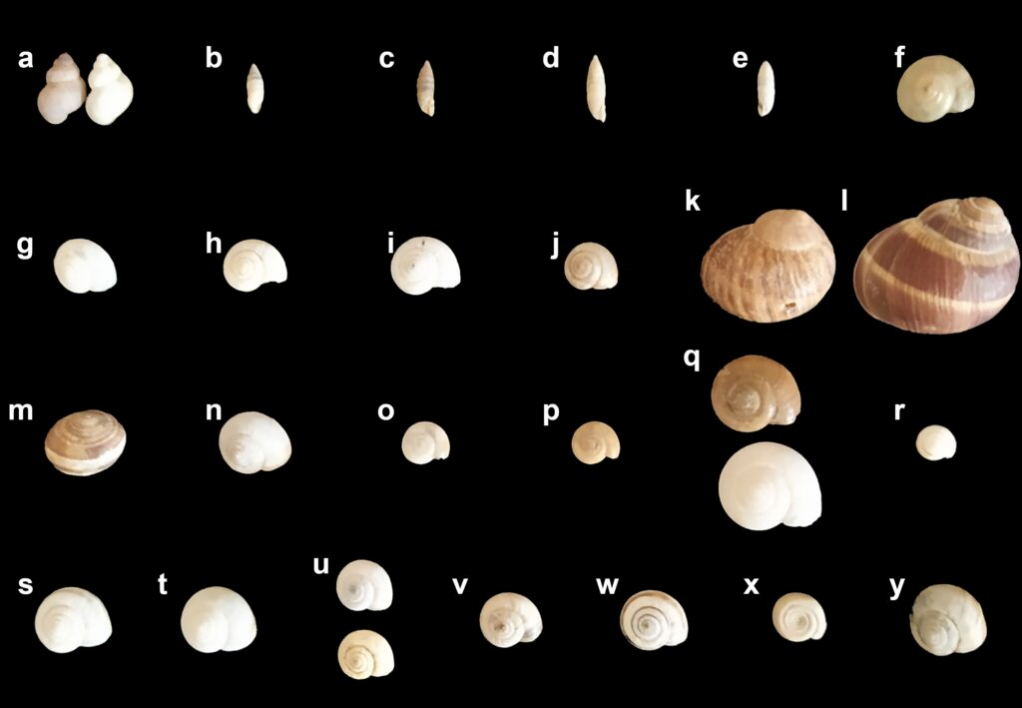
Gastropod fauna of the Pliocene Mountain Spur Natural Reserve. **a** *Pomatiaselegans* (Campiuno); **b** *Granariafrumentumapennina* (Campiuno); **c** *Granariafurmentumillyrica* (Monte Adone); **d** *Granariavariabilis* (Monte Adone); **e** *Jaminiaquadridensquadridens* (road between Monte del Frate and Monte Adone); **f** *Euomphaliastrigellastrigella* (Monte Adone); **g** *Monachacantiana* (Monte Adone); **h** *Monachacartusiana* (Monte del Frate); **i** *Monachamartensiana* (Campiuno); **j** *Monachoidesincarnatus* (Campiuno); **k** *Cornuaspersum* (Monte Mario); **l** *Helixcincta* (Monte Mario); **m** *Helixligata* (Monte Mario); **n** *Morlinaglabraglabra* (Monte Mario); **o** *Oxychilusalliarius* (Monte Mario); **p** Oxychiluscf.draparnaudi (Brento); **q** *Retinellaolivetorumolivetorum* (Fosso Raibano); **r** *Candidulaunifasciataunifasciata* (Monte Mario); **s** *Cernuellaneglecta* (Brento); **t** Cernuellacf.virgata (Fosso Raibano); **u** *Xerogyraspadae* (Monte Mario); **v** *Xerolentaobviaobvia* (Monte Mario); **w** *Xerosectacespitum* (Monte Adone); **x** *Helicodontaobvolutaobvoluta* (Monte del Frate); **y** *Fruticicolafruticum* (Fosso Raibano).

In this work, we report a gastropod fauna comprised of 25 species and subspecies, classified in 18 genera and 10 families (Fig. 3; taxonomy and systematics following WoRMS Editorial Board (2022)). Subfossils shells were found for seven species, i.e. *P.elegans*, *G.frumentumillyrica*, *R.olivetorumolivetorum*, O.cf.draparnaudi, *X.obviaobvia*, *M.cantiana* and *M.cartusiana* (Table 1), which were also found alive. Therefore, there is no evidence pointing to the reduction (neither extinction) of the populations of these species in the Natural Reserve. To our knowledge, this is the first complete report about this mollusc clade in the Tuscan-Emilian Apennine and one of the few in the whole Apennine's mountain chain (De Stefani 1883, Castagnolo and Pettinelli 1993): even the checklist of Italian freshwater gastropod molluscs (Bodon et al. 2005) does not report information from the Pliocene Mountain Spur Natural Reserve.

Still, the present data should be taken as preliminary and further investigation is needed to completely characterise pulmonate molluscs in the Pliocene Mountain Spur Natural Reserve. Taxonomic work is still needed and consensus has not been reached on some of the present names: for example, *Cornuaspersum* (O. F. Müller, 1774) is listed as *Helixaspersa* Müller, 1774 by some authors (for instance, Welter-Schultes (2012)); *Morlinaglabraglabra* (Rossmässler, 1835) is listed as *Oxychilusglaberglaber* (Rossmässler, 1835) by some authors (e.g. Kerney and Cameron (1979)). However, the general figure which is emerging from this site is an assemblage of European and Mediterranean-European elements, with a single Asian species, *Fruticicolafruticum* (Boato et al. 1984, Girod 2011). Furthermore, extensive field research should be carried out to further characterise the gastropod fauna of the Natural Reserve, with special reference to under-represented groups, such as microgastropods or slugs.

Many species are associated with calcareous soils, which result from Pliocene sandstone geochemistry, that commonly present calcareous components (Scarponi 2002). These species are *P.elegans*, *Granariafrumentumillyrica*, *G.variabilis* and *Jaminiaquadridensquadridens*; thus, these species should be taken as good indicators of calcareous soils.

Some taxa are xerothermophilic, meaning that they are associated with xeric areas, which are common in the Reserve because of the microclimate of some sites, such as the base of sandstones walls, which are, in some cases, 100 m high and more (Monte Mario, Rocca di Badolo, Monte Adone). These species are *Xerolentaobviaobvia*, *Euomphaliastrigellastrigella* and *Monachoidesincarnatusincarnatus*, which are definitely xerothermophilic species and should be taken as guide species for these environments, but also *Granariavariabilis*, *Cernuellaneglecta* and Cernuellacf.virgata. *Pomatiaselegans* and *J.q.quadridens* are also associated with, but not exclusive of, xeric environments.

It is of particular interest the discovery of *Helicodontaobvolutaobvoluta*, since it is associated with mountain cool, moist climates. It was collected in a small, moist dell below Monte del Frate along the road to Monte Adone, which, therefore, should be considered as a refugial zone because of its microclimatic conditions.

In broad-leaved, deciduous underbrush and along water bodies, as is the case for the small valley of Fosso Raibano (Fig. 2, site 3), there is a different microclimate, featuring cooler and moister traits. Indeed, some species are associated with that microclimate: *Helixligata*, *Morlinaglabraglabra*, Oxychiluscf.draparnaudi and *Xerogyraspadae*.

Several shells were collected at variables depths (20-60 cm) in sandy-clayey soils and show fossilisation traces, such as the loss of the original colour and the absorption or assimilation of that of the embedding sediment. Therefore, we consider these shells to be subfossil samples (Boato et al. 1984); they were determined as *P.elegans*, *G.f.illyrica*, *Retinellaolivetorumolivetorum*, *Xerolentaobviaobvia*, Oxichiliuscf.draparnaudi, *Monachacartusiana* and *Monachacantiana*.

It is well known (e.g. Raffi and Serpagli (1996)) that granulometry and deposition rate of the sediment significantly affect the fossilisation process that is undergoing within the sediment itself, which can be of many different types. In a nutshell, best preserved fossils are normally expected from fine-grained sediment rather than coarse ones. The organism initially poses on sediment surface and is then embedded, a process which can take place at various speeds. After burial, long chemical and physical processes drive these remains to become fossils; however, remains may be preserved even for long times, without undergoing such modifications - similar cases are known as subfossils (Brouwer 1972).

**Figure 4.**
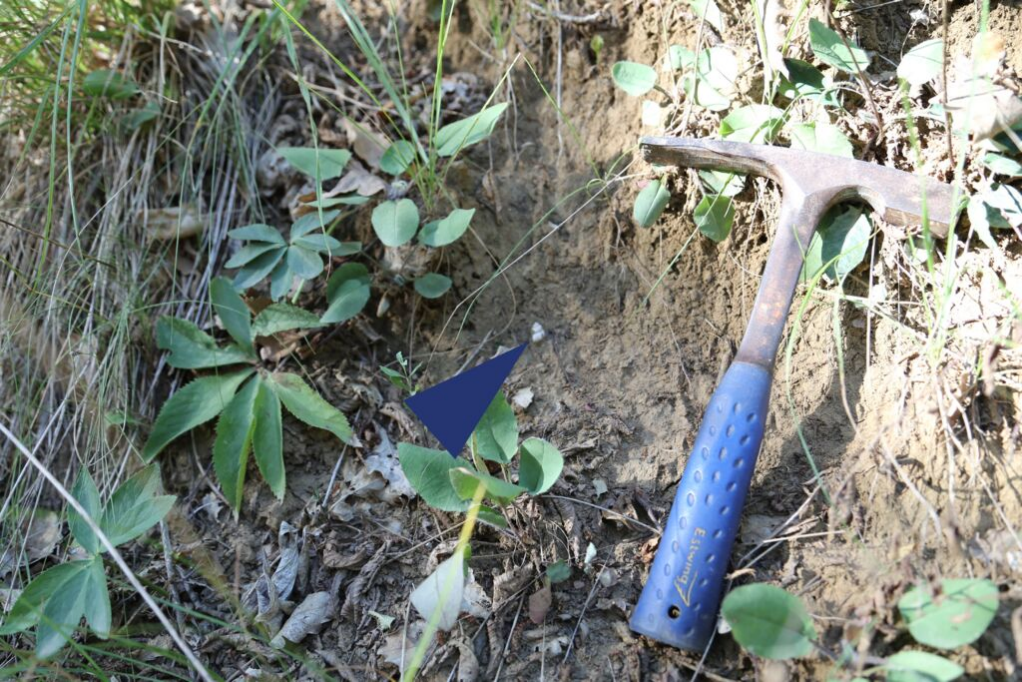
A subfossil specimen of *Pomatiaselegans* (blue arrowhead) in situ (site 7, Campiuno).

Immediately after death, organic matter decomposes; skeletal parts are disjointed, which, for Gastropods, means separating the shell from the operculum; then, maceration can take place in aquatic environments (Raffi and Serpagli 1996). These processes can occupy variable timespans and can lead to prefossilisation, resulting in subfossils. Shells become permineralised, increasing their thickness, or demineralised, reducing in thickness and robustness, because of dissolving substances. The latter phenomenon was observed, for example, in some bivalve fossil specimens from the "great ledge" of Rocca di Badolo. An example of a subfossil specimen of *Pomatiaselegans* is shown *in situ* in Fig. 4.

The conservation status of subfossil specimens is compatible with the appearance of these species in the focal area in the upper Pliocene (end of the Piacenzian, which spans 3.6-2.58 million years ago) or in more recent times (Pleistocene, Holocene), pointing towards a stable presence up to extant populations.

Concluding, the present checklist is the first report in the Tuscan-Emilian Apennine and Emilia-Romagna areas for four taxa: *Morlinaglabraglabra*, *Oxychiliusalliarius*, *Xerosectacespitum* and *Fruticicolafruticum*; moreover, two taxa are reported here for the first time from the entire Northern Apennine (and Emilia-Romagna as well): *Xerogyraspadae* and *Xerolentaobviaobvia*.

## Supplementary Material

XML Treatment for Gastropoda

XML Treatment for Caenogastropoda

XML Treatment for Littorinimorpha

XML Treatment for Littorinoidea

XML Treatment for Pomatiidae

XML Treatment for Pomatias elegans

XML Treatment for Heterobranchia

XML Treatment for Eupulmonata

XML Treatment for Stylommatophora

XML Treatment for Helicina

XML Treatment for Chondrinoidea

XML Treatment for Chondrinidae

XML Treatment for Granaria frumentum apennina

XML Treatment for Granaria frumentum illyrica

XML Treatment for Granaria variabilis

XML Treatment for Pupilloidea

XML Treatment for Enidae

XML Treatment for Jaminia quadridens quadridens

XML Treatment for Helicoidea

XML Treatment for Hygromiidae

XML Treatment for Euomphalia strigella strigella

XML Treatment for Monacha cantiana

XML Treatment for Monacha cartusiana

XML Treatment for Monacha martensiana

XML Treatment for Monachoides incarnatus

XML Treatment for Helicidae

XML Treatment for Cornu aspersum

XML Treatment for Helix cincta

XML Treatment for Helix ligata

XML Treatment for Geomitridae

XML Treatment for Candidula unifasciata unifasciata

XML Treatment for Cernuella neglecta

XML Treatment for Cernuella virgata

XML Treatment for Xerogyra spadae

XML Treatment for Xerolenta obvia obvia

XML Treatment for Xerosecta cespitum

XML Treatment for Helicodontidae

XML Treatment for Helicodonta obvoluta obvoluta

XML Treatment for Camaenidae

XML Treatment for Fruticicola fruticum

XML Treatment for Gastrodontoidea

XML Treatment for Oxychilidae

XML Treatment for Morlina glabra glabra

XML Treatment for Oxychilus alliarius

XML Treatment for Oxychilus draparnaudi

XML Treatment for Gastrodontidae

XML Treatment for Retinella olivetorum olivetorum

## Figures and Tables

**Table 1.** Detected species and relative sampling localities. Sampling sites are shown in Fig. 2. Whenever subfossils shells were found beside living specimens, this is shown in the "Subfossils" column; otherwise, only living specimens were collected.

(Sub)Species	Sampling locality	Elevation (m a.s.l.)	Date	Coordinates	Number of samples	Depth (cm)	Subfossils	Habitat
*Pomatiaselegans*	Fosso Raibano (3)	231	12.IX.2020	44.3711°N, 11.2742°E	> 15	0-60	Yes	Sandy-clayey underbrush
*Pomatiaselegans*	Monte Mario (1)	231	31.VIII.2020	44.3716°N 11.2729°E	> 10	0	No	Grass beside the road
*Pomatiaselegans*	Monte Mario (slope) (2)	241	17.VIII.2021	44.3718°N, 11.2730°E	3	0	No	Sandy underbrush below sandstone cliff
*Pomatiaselegans*	Road to Monte Adone (below Campiuno) (6)	374	30.VIII.2020	44.3479°N, 11.2839°E	> 15	0-60	Yes	Grassy slopes along the road
*Pomatiaselegans*	Campiuno (7)	421	30.VIII.2020	44.3449°N, 11.2888°E	6	0	No	Underbrush slopes with reddish soil
*Pomatiaselegans*	Road between Monte del Frate and Monte Adone (5)	336	28.VIII.2020	44.3482°N, 11.2832°E	8	0	No	Conglomerate outcrops along the road
*Pomatiaselegans*	Monte del Frate (basement) (4)	335	6.IX.2021	44.3511°N, 11.2862°E	> 15	0	No	Very high outcrops above the road
*Granariafrumentumapennina*	Campiuno (7)	421	31.XII.2018	44.3449°N, 11.2888°E	1	0	No	Underbrush slopes with reddish soil
*Granariafrumentumillyrica*	Road between Monte del Frate and Monte Adone (5)	336	28.VIII.2020	44.3482°N, 11.2832°E	2	0	No	Conglomerate outcrops along the road
*Granariafrumentumillyrica*	Campiuno (7)	421	31.XII.2020	44.3449°N, 11.2888°E	1	0-40	Yes	Underbrush slopes with reddish soil
*Granariavariabilis*	Campiuno (7)	421	31.XII.2018	44.3449°N, 11.2888°E	2	0	No	Underbrush slopes with reddish soil
*Jaminiaquadridensquadridens*	Road between Monte del Frate and Monte Adone (5)	336	28.VIII.2020	44.3482°N, 11.2832°E	3	0	No	Conglomerate outcrops along the road
*Jaminiaquadridensquadridens*	Campiuno (7)	421	31.XII.2018	44.3449°N, 11.2888°E	2	0	No	Underbrush slopes with reddish soil
*Euomphaliastrigellastrigella*	Monte Adone (top) (9)	654	13.XII.2021	44.3409°N, 11.2952°E	1	0	No	Sandy plain with sparse shrubs
*Monacacantiana*	Campiuno (7)	421	31.XII.2018	44.3449°N, 11.2888°E	2	0-60	Yes	Underbrush slopes with reddish soil
*Monacacantiana*	Road to Monte Adone (below Campiuno) (6)	374	28.VIII.2020	44.3479°N, 11.2839°E	9, > 15 juv.	0	No	Vegetation beside the road towards Brento
*Monachacartusiana*	Lower road to Monte del Frate, curve (4)	335	28.VIII.2020	44.3511°N, 11.2862°E	2	0-50	Yes	At the base of high sandstone walls
*Monachamartensiana*	Campiuno (7)	421	5.IX.2021	44.3449°N, 11.2888°E	2	0	No	Underbrush slopes with reddish soil
*Monachoidesincarnatus*	Campiuno (7)	421	5.IX.2021	44.3449°N, 11.2888°E	4	0	No	Underbrush slopes with reddish soil
*Cornuaspersum*	Monte Mario (slope) (2)	241	31.VIII.2020	44.3718°N, 11.2730°E	1	0	No	Sandy underbrush below sandstone cliff
*Helixcincta*	Monte Mario (1)	231	31.VIII.2020	44.3716°N, 11.2729°E	> 15	0	No	Grass beside the road
*Helixcincta*	Brento (8)	436	28.VII.2020	44.3373°N, 11.2963°E	3	0	No	Sandstone outcrops
*Helixligata*	Monte Mario (slope) (2)	241	31.VIII.2020	44.3718°N, 11.2730°E	2	0	No	Sandy underbrush below sandstone cliff
*Morlinaglabraglabra*	Monte Mario (slope) (2)	241	31.VIII.2020	44.3718°N, 11.2730°E	1	0	No	Sandy underbrush below sandstone cliff
*Oxychilusalliarius*	Monte Mario (slope) (2)	241	31.VIII.2020	44.3718°N, 11.2730°E	1	0	No	Sandy underbrush below sandstone cliff
Oxychiluscf.draparnaudi	Brento (8)	436	28.VIII.2020	44.3373°N, 11.2963°E	2	0-50	Yes	Sandstone outcrops
*Retinellaolivetorumolivetorum*	Fosso Raibano (3)	231	12.IX.2020	44,3711°N, 11,2742°E	4	0-50	Yes	Sandy-clayey underbrush
*Retinellaolivetorumolivetorum*	Road to Monte Adone (below Campiuno) (6)	241	28.VIII.2020	44.3479°N, 11.2839°E	1	0	No	Vegetation beside the road towards Brento
*Candidulaunifasciataunifasciata*	Monte Mario (slope) (2)	241	17.VIII.2021	44.3718°N, 11.2730°E	2	0	No	Sandy underbrush below sandstone cliff
*Cernuellaneglecta*	Road to Monte Adone (below Campiuno) (6)	241	28.VIII.2020	44.3479°N, 11.2839°E	2	0	No	Vegetation beside the road towards Brento
Cernuellacf.virgata	Fosso Raibano (3)	231	12.IX.2020	44.3711°N, 11.2742°E	1	0	No	Sandy-clayey underbrush
*Xerogiraspadae*	Monte Mario (slope) (2)	241	17.VIII.2021	44.3718°N, 11.2730°E	1	0	No	Sandy underbrush below sandstone cliff
*Xerolentaobviaobvia*	Brento, towards Pianoro (10)	428	5.IX.2021	44.3426°N, 11.3065°E	2	0	No	Grassy sandstone outcrops
*Xerolentaobviaobvia*	Monte Mario (slope) (2)	241	31.VIII.2020	44.3718°N, 11.2730°E	1	0-40	Yes	Sandy underbrush below sandstone cliff
*Xerosectacespitum*	Road between Monte del Frate and Monte Adone (5)	336	12.VI.2022	44.3482°N, 11.2832°E	2	0	No	Conglomerate outcrops along the road
*Helicodontaobvolutaobvoluta*	Lower road to Monte del Frate, curve (4)	335	5.IX.2021	44.3511°N, 11.2862°E	1	0	No	At the base of high sandstone walls
*Fruticicolafruticum*	Fosso Raibano (3)	231	12.IX.2020	44.3711°N, 11.2742°E	1	0	No	Sandy-clayey underbrush
*Fruticicolafruticum*	Brento, towards Pianoro (10)	428	5.IX.2021	44.3426°N, 11.3065°E	3	0	No	Grassy sandstone outcrops
